# Preparation of Topical Itraconazole with Enhanced Skin/Nail Permeability and In Vivo Antifungal Efficacy against Superficial Mycosis

**DOI:** 10.3390/pharmaceutics13050622

**Published:** 2021-04-27

**Authors:** Laxman Subedi, Seung-Yub Song, Saurav Kumar Jha, Sung-Ho Lee, Rudra Pangeni, Kyo-Tan Koo, Beum Joon Kim, Seung-Sik Cho, Jin Woo Park

**Affiliations:** 1 Department of Biomedicine, Health & Life Convergence Sciences, BK21 Four, Biomedical and Healthcare Research Institute, Mokpo National University, Jeonnam 58554, Korea; laxmansubedi789@gmail.com (L.S.); tgb1007@naver.com (S.-Y.S.); saurav.balhi@gmail.com (S.K.J.); tjdgh0730@naver.com (S.-H.L.); 2 Department of Pharmacy, College of Pharmacy and Natural Medicine Research Institute, Mokpo National University, Jeonnam 58554, Korea; capriconpangeni@gmail.com; 3 BioBelief Co., Ltd., Seoul 05841, Korea; kyotankoo@gmail.com; 4 Department of Dermatology, Chung-Ang University College of Medicine, Seoul 06973, Korea; beomjoon74@gmail.com

**Keywords:** itraconazole, topical solution, superficial mycosis, skin penetration, nail infiltration, antifungal activity

## Abstract

In this study, a stable and highly skin-permeable topical delivery system for itraconazole (ITZ) was designed to provide effective treatment against superficial mycosis. Herein, ITZ was incorporated into a solution composed of ethanol, benzyl alcohol, hydrochloric acid, Transcutol P, and cyclomethicone as a delivery vehicle, solubilizer, protonating agent, permeation enhancer, and spreading agent, respectively. At 72 h, the optimal topical ITZ formulation (ITZ–TF#11) exhibited 135% enhanced skin permeability, which led to increases in drug deposition in the stratum corneum, epidermis, and dermis of 479%, 739%, and 2024%, respectively, compared with the deposition of 1% ITZ in ethanol (control). Moreover, on day 7, ITZ–TF#11 demonstrated 2.09- and 2.30-fold enhanced nail flux and drug deposition, compared with the control. At a dose of 40 mg/kg/day, ITZ–TF#11 showed 323% greater lesion recovery, a 165% lower mean erythema severity score, and a 37% lower mean logarithm of viable fungal cells in skin in the treated area, compared with mice that received oral ITZ at the same dose. Overall, the findings imply that ITZ–TF#11 is a superior alternative to oral ITZ for treatment of superficial mycosis.

## 1. Introduction

Superficial mycosis is a fungal disease that is mostly confined to outer layers of skin, nail, or hair but may invade deeper tissue. This disease is common worldwide [1]. Moreover, mycosis has been reported to negatively impact host psychological and psychosocial factors and reduce quality of life [2]. Various types of wide-spectrum antifungal azoles (e.g., efinaconazole [EFN], fluconazole, ketoconazole, voriconazole, and itraconazole [ITZ]) are available for use in mycosis treatment. Importantly, azole groups interfere with the synthesis of ergosterol, an essential fungal membrane component, by blocking 14-α demethylase (a cytochrome P-450 enzyme). In addition, the selectivity of azole groups toward the cytochrome P-450 enzymes of fungi, rather than mammals, enables highly specific antifungal action with comparatively minimal side effects, relative to other antifungal treatments [3]. Among several azole-based antifungal agents, 2-butan-2-yl-4-[4-[4-[4-[[2-(2,4-dichlorophenyl)-2-(1,2,4-triazol-1-ylmethyl)-1,3-dioxolan-4-yl] methoxy] phenyl]-1-piperazinyl] phenyl]-1,2,4-triazol-3-one (ITZ) has strong keratinophilic and lipophilic properties and has therefore emerged as an effective broad-spectrum antifungal treatment for superficial mycosis [4]. Furthermore, ITZ has been used successfully in the treatment and prevention of *Aspergillus* infections, with fewer toxic side effects than amphotericin B, indicating a better therapeutic index [5].

Clinical treatment of cutaneous mycosis with ITZ generally involves oral administration. Despite considerable absolute oral bioavailability (approximately 70%), an absence of blood vessels in the epidermis and stratum corneum can limit the availability of ITZ at the desired site of action [6,7]. In addition, because of its large molecular size (705.64 Da) and enhanced plasma-binding affinity, ITZ may not effectively diffuse from the dermis sublayer to other layers of the epidermis and stratum corneum [8]. Thus, greater concentrations of ITZ have been achieved with typical oral doses of 200–400 mg/day [9,10]. However, the resulting high systemic concentration of ITZ has been reported to cause nephrotoxicity and hepatic damage, as well as various reproductive complications [11]. Therefore, topical administration of ITZ can be advantageous for site-specific delivery, with fewer side effects than oral administration.

Approaches such as creams, ointments, pastes, and liposomal formulations have been used to improve topical delivery of ITZ [12]. Colloidal carrier systems incorporating ITZ can moderately enhance its solubility. However, the semi-solid nature of colloidal carriers minimizes drug release and reduces formulation spreading on the skin surface. It also leads to limited permeability across the skin membrane [13]. Furthermore, residues of particulate carriers on skin after the application of the above formulations may reduce patient compliance. Therefore, incorporation of volatile vehicles may be a suitable alternative approach in topical ITZ solution development. Additionally, the volatile vehicle used in topical formulations has been reported to follow the metamorphism phenomenon, which leads to drug supersaturation within the vehicle system at the applied area over time, which can increase both effective and immediate drug delivery [14]. The incorporation of a permeation enhancer, which interacts with lipid and protein constituents present in the outermost layer of skin, can increase drug penetration and partitioning [15]. Therefore, the generation of a stable ITZ solution with a volatile vehicle, effective permeation enhancer, and wetting agent to promote spreading may enhance the clinical effects of ITZ on superficial mycosis.

This study sought to design a topical delivery system for ITZ with enhanced solubility, robust skin penetration, and longer storage stability and to then compare the in vivo antifungal efficacy of this new system with a commercially available oral formulation. Herein, to prepare a topical ITZ formulation (ITZ–TF), ethanol and benzyl alcohol were used as a volatile vehicle and solubilizing agent, respectively. To enhance the solubility and stability of ITZ in solution, hydrochloric acid was used as a protonating agent and sodium hydroxide was incorporated to maintain the solution pH within the physiological range of skin (pH 4.5–5.5). This pH range was presumed to aid in maintenance of stability after application to skin and to promote effective partitioning of ITZ across the skin with fewer side effects [14]. Furthermore, Transcutol P and cyclomethicone were used as a permeation enhancer and spreading agent, respectively. The permeabilities of the new ITZ formulation across the artificial membrane, human skin, and human nail were examined. Based on the permeability results, the optimal formulation was subjected to comparative in vivo antifungal efficacy testing at a dose identical to that of commercially available oral ITZ, using a BALB/c mouse model of dermal *Candida albicans* infection.

## 2. Materials and Methods

### 2.1. Materials

ITZ was obtained from SVR Drugs Pvt Ltd. (Telangana, India). Polyoxyl-40 hydrogenated castor oil (Kolliphor RH 40), polyoxyl-35 castor oil (Cremephor EL), cyclomethicone, butylated hydroxyanisole (BHA), ethylenediamine tetraacetic acid (EDTA), and benzyl alcohol were purchased from Sigma-Aldrich Inc. (St. Louis, MO, USA). Diethylene glycol monoethyl ether (Transcutol P) was obtained from Gattefossé (Saint Priest, France). Ethanol, sodium hydroxide, and hydrochloric acid were purchased from Reagents Duksan (Gyeonggi-do, Korea). Sabouraud dextrose agar was obtained from BD Biosciences (San Jose, CA, USA). ITZ oral capsule (reference control for in vivo study) was provided by Janssen Korea Ltd. (Seoul, Korea). EFN (internal standard for liquid chromatographic mass spectroscopy [LC/MS] analysis) was obtained from Cipla Limited (Mumbai, India). Reagents such as acetonitrile and formic acid, which were required to prepare the mobile phase, were obtained from Sigma-Aldrich Inc. All chemical reagents used in this study were of analytical grade. All solvents were of high-performance liquid chromatography (HPLC) grade.

### 2.2. Animals

Female BALB/c mice (6 weeks old, 20 g) were purchased from OrientBio (Gwangju, Korea). All mice were housed in a controlled environment in terms of temperature (23 ± 2 °C), relative humidity (55 ± 10%), and light (12/12-h light/dark cycle) throughout the duration of the experiment. In addition, animals were provided free access to a standard laboratory diet (Bestle Purina PetCare Research, St. Louis, MO, USA) and ion-sterilized water. Ethical approval for this study protocol was obtained from the Institutional Animal Care and Use Committee of Mokpo National University (Jeonnam, Korea: approval no. MNU-IACUC-2020-020). All experiments were performed in accordance with the National Institutes of Health guidelines for the Care and Use of Laboratory Animals and the guidelines of the institutional animal care and use committee.

### 2.3. Solubility of ITZ in Topical Formulations

The major impediment to skin permeation of ITZ is its low aqueous solubility [16]. Therefore, various solvents (e.g., ethanol, benzyl alcohol, Transcutol P, Cremephor EL, Capryol 90, polyethylene glycol 400, oleic acid, isopropyl myristate, tetraethylene glycol, and n-methyl-2-pyrrolidone) were selected and subjected to screening for their ability to enhance ITZ solubility. An excess amount of ITZ was initially added to each individual solvent and mixed using a vortex mixer at room temperature. After 48 h, samples were centrifuged at 10,000 rpm for 10 min and the supernatant was diluted with a mobile phase consisting of acetonitrile and water (pH adjusted to 4.0 with phosphoric acid) (80:20, *v*/*v*). The concentration of ITZ in each diluted sample was measured via HPLC at 263 nm. A Luna C18 column (250 × 4.6 mm, 5-µm particle size, 20-µL injection volume) was used with a mobile phase at a flow rate of 0.7 mL/min.

### 2.4. Preparation and In Vitro Artificial Membrane Permeability of Topical ITZ Formulation

To formulate 1% ITZ topical formulation (ITZ–TF; 1 g ITZ/100 g of solution), benzyl alcohol and ethanol were selected as a solubilizing agent and delivery vehicle, respectively. To provide additional stability, BHA was incorporated as an antioxidant and EDTA was incorporated as a chelating agent (Table 1). Briefly, 0.05 g of ITZ was weighed in a glass vial and dissolved by using 0.5, 1.0, and 1.5 g of benzyl alcohol, respectively. Then, 0.005 g of BHA and 0.05 g of aqueous solution of EDTA (0.025 mg/mL) were added and vortex mixing was performed. Hydrochloric acid was then added at concentrations of 0.121%, 0.242%, and 0.484% into the topical formulations for the protonation of ITZ, thereby enhancing its solubility and stability. Subsequently, 1.5 g of ethanol was incorporated and mixed for 1 min. To improve the permeability of ITZ across the skin and nails, various amounts of Transcutol P (10%, 15%, and 20%) and cyclomethicone (5.00% and 8.22%) were added to the formulations. Sodium hydroxide was added to adjust the pH to the range 4.5–5.5. Finally, the weight of the formulation was maintained at 100% by addition of ethanol. The effects of multiple concentrations of various excipients on the solubility and miscibility of ITZ, and on other components in the ITZ–TF system, were evaluated. Moreover, changes in drug concentration and physiochemical properties (e.g., color, precipitation, and phase separation) were examined weekly for >3 months.

The in vitro artificial membrane permeability of ITZ–TF was investigated using a synthetic membrane (Strat-M^®^; EMD Millipore, Temecula, CA, USA) and a Franz diffusion cell system (Labfine, Gyeonggi-do, Korea). In this analysis, each membrane was sandwiched between the donor and receptor compartments with a 0.785-cm^2^ diffusion area. The receptor compartment was filled with 5 mL of phosphate-buffered saline (PBS; pH 7.4) containing 15% acetonitrile as a receptor-phase solution to maintain the sink condition [17]. The solution in the receptor cell was stirred using a magnetic stirrer at 600 rpm within a heating system to maintain the membrane surface temperature at 32 °C throughout the experiment. Afterwards, 50 µL of each formulation (636 µg/cm^2^) was loaded into the donor compartment. Then, 500-µL aliquots of the receptor phase were withdrawn from the sampling port at pre-determined time intervals (0.5, 1, 1.5, 2, 3, 4, 5, and 6 h) and replaced with equivalent volumes of fresh receptor solution. Collected samples were filtered through a membrane filter (polyvinylidene fluoride [PVDF] with 0.45-µm pore size) and analyzed using an HPLC system at 263 nm as described above.

### 2.5. In Vitro Permeation and Deposition of ITZ in Full-Thickness Human Skin

To compare the human skin permeabilities of ITZ in each vehicle, excised full-thickness human skin (HansBiomed Corp., Daejeon, Korea) was mounted (with the stratum corneum upward) over the receptor compartments of a Franz diffusion cell filled with 5 mL PBS (pH 7.4): acetonitrile 85:15 (*v*/*v*). The solubility of ITZ in this solution was 1.58 ± 0.10 mg/mL, thus 15.8-fold higher than the maximum concentration of ITZ (100 µg/mL) in the receptor compartment; the sink condition was met [17]. Prior to addition of test samples to donor cells, the receptor cells were allowed to equilibrate for at least 1 h and only skin samples exhibiting transepidermal water loss (TEWL) < 10 g/m^2^/h and transepithelial electrical resistance (TEER) between 1–2 kΩ were used; this guaranteed the integrity of the epidermal membrane [18]. The receptor compartment was stirred at 600 rpm to maintain the skin surface temperature at 32 °C throughout the experiment. Then, 50 µL amounts of each 1% ITZ formulation (636 µg/cm^2^) were loaded into the donor compartments at 24 h intervals. Five hundred-microliter aliquots of the receptor phase were withdrawn from each diffusion cell at 0, 0.5, 1, 1.5, 2, 3, 4, 5, 6, 8, 10, 24, 48, and 72 h and replaced with equivalent volumes of fresh receptor solution. All samples were passed through PVDF membrane filters (0.45-µm in pore diameter) and analyzed using the HPLC method described above.

To evaluate ITZ skin deposition after completion of the full-thickness human skin permeability study, the skin sections were rinsed five times with distilled water and gently wiped with tissue paper to remove residual formulations. The skin sections were weighed to measure initial weight. To isolate the stratum corneum, the skin was stripped 35 times with D-squame^®^ (CuDerm, Dallas, TX, USA) [19]. The stratum corneum weights were calculated by subtracting the weights after stripping from the initial weights. Next, the epidermis and dermis were thermally separated via immersion in distilled water at 60 °C for 1 min. The epidermis was then carefully isolated from the dermis using a pair of forceps. The stripped tape containing stratum corneum, epidermis, and dermis was cut into small pieces and immersed in 5 mL of methanol and then extracted via vortex mixing for 24 h [13]. The resulting suspensions were filtered using membrane filters (0.45-µm pore size) and transferred to glass vials. Internal standard (100 µL of 5 µg/mL EFN) was added and the mixture was dried using a rotary evaporator. Thereafter, the dried residue was reconstituted in 200 µL of a 1:1 (*v*/*v*) mixture of methanol and mobile phase (acetonitrile:0.1% formic acid in water, 60:40 [*v*/*v*]) and subjected to LC/MS analysis. Finally, the concentration of ITZ in the reconstituted sample was determined using an Agilent 6120 quadruple LC/MS system with a Luna C18 column (250 × 4.6 mm, 5 µm) as a stationary phase. Aliquots (20 µL) from each sample were eluted in the mobile phase at a flow rate of 1 mL/min. ITZ and EFN were ionized using an electrospray ionization source in the positive-ion mode under the following conditions: capillary voltage of 4 kV, drying gas flow rate of 10.0 L/min, and drying temperature of 100 °C. Quantitative analyses of protonated molecular ions were performed at ([M + H]^+^ = 706.8) and ([M + H]^+^ = 349.4), for ITZ and EFN, respectively.

### 2.6. In Vitro Human Nail Permeability and Deposition of ITZ

To examine the infiltration of ITZ across the human nail, in vitro nail permeation and deposition analyses were performed using a Franz diffusion cell system. Human nails obtained from Science Care, Inc. (Phoenix, AZ, USA) were stored at –80 °C until use. One day before the experiment, the nails were transferred to room-temperature storage conditions. On the day of the experiment, nail plates were stored in normal saline to ensure sufficient hydration. They were then fixed between two Teflon nail-holder gaskets, which were mounted between the donor and receptor compartments. Receptor solution (5 mL; PBS [pH 7.4]:acetonitrile, 85:15, [*v*/*v*]) was then used to fill the receptor compartment. The solution in the receptor compartment was stirred at 600 rpm to maintain a nail surface temperature of 32 °C throughout the experiment. The respective 1% ITZ–TF with a once-daily dose (20 µL per dose) was then loaded into the donor compartment for 7 days. Aliquots (500 µL) of the receptor phase were withdrawn at intervals of 1, 2, 3, 4, 5, 6, and 7 days and replaced with equivalent volumes of fresh receptor solution. The withdrawn samples were filtered with a membrane filter (0.45-µm PVDF). Afterwards, 100-µL aliquots of filtrate samples were mixed with 100 µL of EFN drug solution (5 µg/mL, internal standard) in methanol and analyzed via LC/MS, as mentioned above.

To monitor ITZ deposition in the nail, diffusion cells were disassembled and nail plates were isolated. All nail plates were washed twice with 5 mL of water. Afterwards, the area effectively exposed to the drug formulation was cut into small pieces and dissolved in 1 M sodium hydroxide:methanol (1:1, *v*/*v*) solution. The nail suspensions were incubated at 60 °C for 4 h and then mixed with 1 mL of methanol and 1 mL of ethanol in conjunction with continuous vortex mixing [20]. After incubation had been completed, the nail suspension was centrifuged at 4000 rpm for 10 min to obtain clear supernatant, which was then mixed with 100 µL of internal standard (5 µg/mL EFN) in a glass vial. The mixture of supernatant and internal standard was evaporated using a rotatory evaporator (Genevac Ltd., Ipswich, UK). Finally, the dried residue was reconstituted in 200 µL of a 1:1 (*v*/*v*) mixture of methanol and mobile phase (acetonitrile:0.1% formic acid in water, 60:40, [*v*/*v*]) and analyzed via LC/MS, as described above.

### 2.7. In Vitro Antifungal Efficacy

The susceptibility of *C. albicans* to various ITZ samples was evaluated via the disc diffusion method. At first, a *C. albicans* (100 µL, 10^7^ colony-forming unit, CFU) was loaded onto potato dextrose agar. Next, paper discs (8 mm, Toyo, Japan) soaked in ITZ sample (40 µL, 100 µg/mL concentration of ITZs) were arranged on a potato dextrose agar plate and then incubated for 24 h at 28 °C. Finally, zones of inhibition were calculated by measuring the mean diameter of the clear area around each ITZ paper disc.

The in vitro minimum inhibitory concentration (MIC, µg/mL) of ITZ was determined via serial dilution in potato dextrose medium. *C. albicans* (10^7^ CFU/mL) was exposed to six different concentrations of ITZ (0, 3.125, 6.25, 12.5, 100, and 200 µg/mL) at 28 °C for 24 h. Then, MIC was determined visually and spectrophotometrically at 660 nm. After the MIC test, 100 µL of culture broth containing ITZs was loaded onto Sabouraud dextrose agar plates and incubated at 28 °C for 48 h. The in vitro fungicidal activity (MFC) value was determined using the lowest drug concentration that showed 100% growth inhibition of the fungal strains.

### 2.8. In Vivo Skin Irritation and Antifungal Efficacy of ITZ–TF

The in vivo skin irritation and antifungal potentials of ITZ–TF were evaluated using BALB/c mice. The mice were randomly divided into six groups (groups 1–6, *n* = 15 mice per group), as shown in Table 2. Because of the opportunistic nature of *C. albicans* in immunocompromised hosts, mice in groups 3, 4, 5, and 6 underwent immunosuppression using dexamethasone sodium phosphate in water at a dose of 5 mg/kg/day through the intraperitoneal route for 3 days. On the penultimate day of immunosuppression, approximately 2 × 2 cm^2^ of dorsal hair was shaved off all mice using an electric clipper. Afterwards, a scratch of approximately 1.75 × 1.75 cm^2^ was made on the shaved area in mice from groups 3, 4, 5, and 6 using sterile fine-grit sandpaper (120 mesh). To induce fungal infection, 100 µL of *C. albicans* (10^7^ CFU) was continuously applied to the shaved and lightly scarified skin of mice in groups 4, 5, and 6 for 3 days. The induction of fungal infection in groups 4, 5, and 6 was confirmed by comparison of clinical scores (based on the presence of exudate, erythema, and a white substance over the infected area) with the findings in group 3. The symptoms were individually scored as follows: 0 (complete absence), 1 (slightly visible), 2 (clearly visible), 3 (substantially visible in a small area), and 4 (substantially visible in a large area). The mean severity score of each symptom was calculated separately for each group. Moreover, to evaluate antifungal efficacy, the lesion area was measured by means of a digital camera and ImageJ software (LOCI); the healing rate was expressed as a proportion of the remaining wound area. Additionally, skin sections (approximately 2 × 2 cm^2^) from the infected sites of 20 randomly selected mice (*n* = 5 mice per group; groups 3, 4, 5, and 6) were excised for mycological evaluation. The skin samples were punched at three different sites to obtain subsamples 8 mm in diameter. Each subsample was vortexed in Sabouraud dextrose medium (without antibiotics) for 1 min to extract all remaining viable fungi. Then, each extract was loaded onto a Sabouraud dextrose agar plate and incubated at 37 °C. After 48 h, colonies were counted and mean values (CFUs/1 cm^2^ skin) calculated. Finally, via comparisons of the severity scores and mycological statuses of mice from groups 4, 5, and 6 with those from group 3, fungal infection was confirmed. Treatment was then initiated for 10 days using ITZ samples in mice from all groups. Treatment of mice with ITZ samples at the dose equivalent to 40 mg/kg/day was performed as follows: groups 1, 3, and 4 [ITZ–TF#11 (vehicle)], groups 2 and 3 [ITZ–TF#11], and group 5 [ITZ (oral)].

To examine the skin-irritation and antifungal-efficacy characteristics of each ITZ sample, continuous severity evaluation was performed throughout treatment. On the 11th day of treatment, all mice were sacrificed and their skins were isolated for further analysis. Mycological evaluation was performed as described above to directly access the antifungal activities of the ITZ samples. Antifungal efficacy at the site of infection was evaluated via histopathological assay via hematoxylin and eosin (H&E) and periodic acid–Schiff (PAS) staining. For H&E staining, the skin was blocked using paraffin and stained with H&E dye to enable microscopic examination and characterization of inflammatory patterns, blood vessels, and exudate. For PAS staining, skin specimens were rinsed three times with PBS and fixed overnight in Carnoy’s solution. Skin specimens were then immersed in sterile PBS, washed and processed for dehydration with ethanol, vitrified with xylene, and immersed and embedded in paraffin. Microscopic examination of skin was performed to visualize bright reddish-pink color indicating carbohydrate analogs present in the fungal cell wall or cell capsule [21].

### 2.9. Statistical Analyses

All data are expressed as means ± standard deviations. Unpaired data were evaluated using Student’s *t*-test for comparisons between two mean values, and one-way analysis of variance followed by Tukey’s multiple-comparison test was performed for comparisons among more than two mean values. In all analyses, *p* < 0.05 was considered statistically significant.

## 3. Results and Discussion

### 3.1. Drug Stability and In Vitro Artificial Membrane Permeability of ITZ–TF

To develop an effective topical ITZ formulation (ITZ–TF) with enhanced solubility–permeability, a biphasic system composed of volatile and non-volatile components was designed. First, based on its safety profile, ethanol was selected as a delivery vehicle. BHA, EDTA, and 1 M sodium hydroxide were used as an antioxidant, chelating agent, and pH-adjusting agent, respectively, for additional stability. Next, based on the solubility of ITZ, benzyl alcohol was used as a co-solvent. After the addition of benzyl alcohol at 10%, 20%, and 30% concentrations in ITZ–TF#1, ITZ–TF#2, and ITZ–TF#3, respectively, approximately 29-fold greater drug concentrations were achieved in the solubilized form than in 1% ITZ in ethanol (control solution) on day 0 (Table 3). In addition, the artificial membrane permeability of ITZ–TF#3 was enhanced by 12.9-fold, compared with the control (Table 3). However, on day 30, ITZ–TF#3 demonstrated 69.7% drug content, which suggested precipitation of the drug through an anti-solvent crystallization phenomenon due to the presence of polar solvents (e.g., ethanol and water) that enable low ITZ solubility [22]. Thus, to prevent drug precipitation, hydrochloric acid at concentrations of 0.121%, 0.242%, and 0.484% was added to ITZ–TF#4, ITZ–TF#5, and ITZ–TF#6, respectively. The solubility and stability in the vehicle with a polar system were thus achieved via drug protonation and via the formation of a stable complex between ITZ and hydrochloric acid [23]. The results showed that ITZ–TF#6 exhibited a 99.5% drug content until day 30 (Table 3). However, the drug permeability of ITZ–TF#6 was not enhanced, compared with that of ITZ–TF#3. To enhance the permeability, we incorporated 10%, 15%, and 20% concentrations of Transcutol P in ITZ–TF#7, ITZ–TF#8, and ITZ–TF#9, respectively. The artificial-membrane drug permeability of ITZ–TF#7, ITZ–TF#8, and ITZ–TF#9 was enhanced by 2.89-, 4.08-, and 4.11-fold, respectively, compared with that of ITZ–TF#6. The drug contents of those formulations were maintained at approximately 99% until day 30 (Table 3). However, the almost identical drug penetration properties of ITZ–TF#8 and 9 suggested that 15% Transcutol P provided maximal enhancement of drug permeability, presumably due to the saturation of Transcutol P to maintain the necessary thermodynamic driving force for effective flux across the barrier membrane [24]. Furthermore, 5% and 8.22% concentrations of cyclomethicone as a wetting agent were added to ITZ–TF#10 and 11, respectively. ITZ–TF#11 then showed 2.35-fold greater drug permeability, compared with ITZ–TF#8, which may be due to the enhancing effect of cyclomethicone on drug infiltration by lowering surface tension and increasing the spreadability of topical formulations (Table 3) [20]. Moreover, ITZ–TF#11 was able to maintain the drug content within the range of 95.0–105.0% for more than 90 days, and no changes in physical appearance (e.g., precipitation, phase separation, or color change) were observed.

### 3.2. In Vitro Permeation and Deposition of ITZ in Full-thickness Human Skin

**Figure 1:**
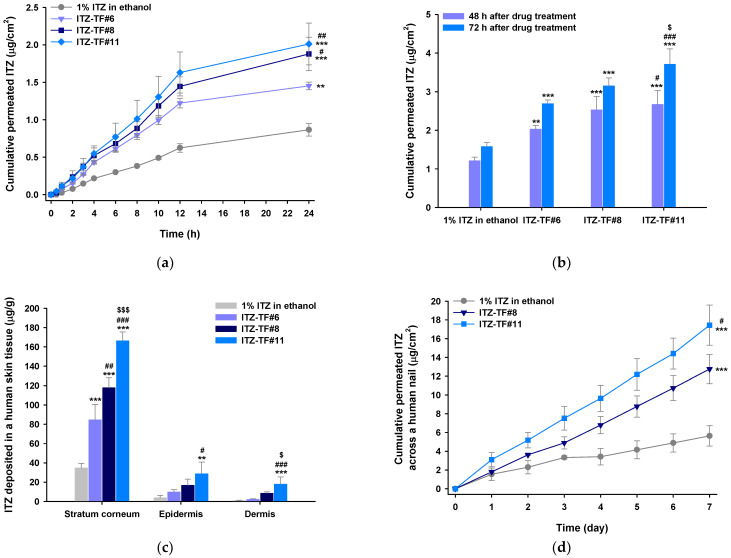
The in vitro full-thickness human skin and nail permeabilities of itraconazole (ITZ) from 1% ITZ in ethanol, ITZ–TF#6, ITZ–TF#8, and ITZ–TF#11. (**a**) The cumulative ITZ levels that permeated human skin over 24 h. (**b**) Cumulative human skin ITZ infiltrations at 48 and 72 h. (**c**) ITZ concentrations/g of stratum corneum, epidermal, and dermal tissues. (**d**) The time course of cumulative, partitioned drug concentrations across human nails from day 1 to 7. Each value is a mean ± standard deviation (*n* = 4). ** *p* < 0.01, *** *p*  <  0.001 compared to 1% ITZ in ethanol. ^#^ *p* < 0.05, ^##^ *p* < 0.01, ^###^ *p* < 0.001 compared to ITZ–TF#6. ^$^ *p* < 0.05, ^$$$^ *p* < 0.001 compared to ITZ–TF#8.

The skin permeations and deposition levels of ITZ from 1% ITZ in ethanol, ITZ–TF#6, ITZ–TF#8, and ITZ–TF#11 were determined using the Franz diffusion system. ITZ from ITZ–TF#6, ITZ–TF#8, and ITZ–TF#11 was detected in the receptor phase after 0.5 h of permeation. However, skin permeation of ITZ from 1% ITZ in ethanol commenced at only 1.5 h (Figure 1a). The cumulative permeated ITZ level from ITZ–TF#6 was 1.45 ± 0.051 µg/cm^2^ at 24 h, thus 67% greater than the value from 1% ITZ in ethanol. Such permeability enhancement may be attributable to the 29.8-fold greater drug concentration of ITZ–TF#6 compared to that of 1% ITZ in ethanol on day 0. In addition, skin drug penetration from ITZ–TF#8 was more rapid than that from ITZ–TF#6; the 24 h cumulative permeated drug level from ITZ–TF#8 was 29.3% higher than that from ITZ–TF#6. This may reflect an effect of Transcutol P, which creates stratum corneum channels via lipid extraction from the bilayer matrix, fluidization of the lipid bilayer, changes in stratum corneum protein conformations, and creation of a concentration gradient that drives drug permeability. Furthermore, amphiphilic Transcutol P enhances drug solubility in lipids of the stratum corneum, and prevents precipitation of lipophilic drugs in water during skin penetration [24]. The permeated drug levels from ITZ–TF#11 were 7.00% and 28.0% greater than those from ITZ–TF#8 at 24 and 72 h, respectively (Figure 1b). This may reflect the presence of cyclomethicone in ITZ–TF#11, unlike ITZ–TF#8. Cyclomethicone enhances skin moisture retention and, in synergy with Transcutol P, promotes intercellular lipid fluidization in the stratum corneum.

The ITZ deposition levels in various skin layers after application of 1% ITZ in ethanol, ITZ–TF#6, ITZ–TF#8, and ITZ–TF#11 over 3 days are shown in Figure 1c. The deposition levels were highest in the stratum corneum, followed by the epidermis and dermis, presumably because of the lipophilic nature of ITZ, which should bind readily to lipids of the stratum corneum [25,26]. ITZ–TF#6 dermal deposition was 2.43-fold greater than dermal deposition from the 1% ITZ in ethanol. The ITZ–TF#8 drug depositions in the stratum corneum, epidermis, and dermis were 210%, 169%, and 399% those of ITZ–TF#6. The 2.11-fold greater dermal drug deposition of ITZ–TF#11, compared to ITZ–TF#8, may reflect enhanced drug partitioning of ITZ–TF#11 across human skin.

### 3.3. In Vitro Human Nail Permeability and Deposition

The permeation potential across the nail plates of ITZ in 1% ITZ in ethanol, ITZ–TF#8, and ITZ–TF#11 was evaluated as shown in Figure 1d. After 24 h of drug loading, the permeability of ITZ from ITZ–TF#8 was 1.80 ± 0.093 µg/cm^2^, which was 117% greater than the permeability of ITZ from 1% ITZ in ethanol (Figure 1d). In addition, ITZ–TF#8 demonstrated 226% greater cumulative permeated drug concentration, compared with 1% ITZ in ethanol, on day 7. These enhanced permeabilities of ITZ–TF#8 may have been due to the greater drug concentration in solubilized form in ITZ–TF#8, as well as the presence of bipolar Transcutol P in ITZ–TF#8, which can interact with both hydrophilic and lipophilic components present in the nail matrix [27,28]. Moreover, on day 7, ITZ–TF#11 produced a 36.7% enhancement of drug penetration across the nail, compared with ITZ–TF#8. This enhancement may have been due to the presence of cyclomethicone, which lowers the surface tension of ITZ–TF#11 and accelerates drug penetration from the small channels generated after conformational changes in the nail keratin structure caused by Transcutol P [29,30]. Furthermore, after continuous application for 7 days, 1% ITZ in ethanol, ITZ–TF#8, and ITZ–TF#11 showed 31.4 ± 5.68, 42.9 ± 4.23, and 72.2 ± 14.9 µg/g deposition of the drug in the nail plate, respectively. Thus, based on the comparative enhanced nail penetration and deposition of the drug in nail plates, ITZ–TF#11 was identified as the optimal formulation, compared with 1% ITZ in ethanol and ITZ–TF#8.

### 3.4. In Vitro Antifungal Activity

**Figure 2:**
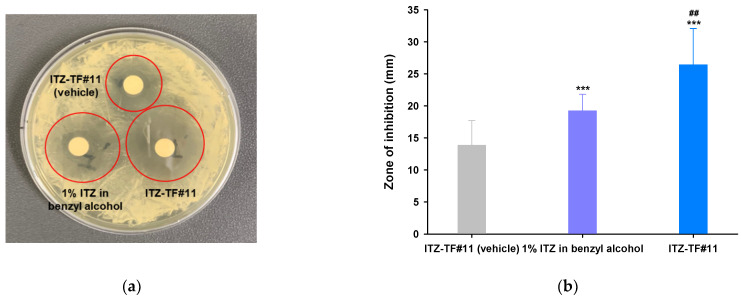
In vitro antifungal efficacies of ITZ–TF#11 (vehicle), 1% ITZ in benzyl alcohol, and ITZ–TF#11. (**a**) Image depicting zones of inhibition of *C. albicans* in a Petri dish. (**b**) Zone of inhibition (mm). Each value represents the mean ± standard deviation (*n* = 4). *** *p* < 0.001 compared with ITZ–TF#11 (vehicle). ^##^ *p* < 0.01 compared with 1% ITZ in benzyl alcohol.

The potential of a formulation to inhibit the growth of fungal cells effectively is known as its fungicidal activity. This fungicidal effect is mediated by its solubility and permeability across the fungal cellular membrane. Although ITZ has considerable antifungal capability, its poor solubility and stability are important obstacles that restrict permeability across the fungi cell membrane and substantially reduce its in vitro antifungal effect. Based on its solubility, stability, and enhanced in vitro permeability across human skin and nail, we selected ITZ–TF#11 to evaluate its in vitro antifungal effects against *C. albicans*. In addition, we used ITZ–TF#11 (vehicle) and 1% ITZ in benzyl alcohol for comparison. As shown in Figure 2, the mean zones of inhibition of ITZ–TF#11 were 244% and 163% greater than the mean zones of inhibition of ITZ–TF#11 (vehicle) and 1% ITZ in benzyl alcohol, respectively. Moreover, MFCs of both ITZ–TF#11 and 1% ITZ in benzyl alcohol were 4-fold lower than the MFC of ITZ–TF#11 (vehicle). The MIC of ITZ–TF#11 was 4- and 10.6-fold lower than the MICs of 1% ITZ in benzyl alcohol and ITZ–TF#11 (vehicle), respectively (Table 4). Such enhanced fungicidal activity of ITZ–TF#11 against *C. albicans* may reflect increased drug permeation mediated by Transcutol P and rapid drug penetration facilitated by the wetting agent (cyclomethicone). They also enhance drug cellular uptake via endocytosis and passive drug diffusion into the cytoplasm; Transcutol P and cyclomethicone increase lipid extraction and fluidization of the lipid bilayer and improve the partition coefficients of drugs relative to those of cell membranes [31,32,33].

### 3.5. In Vivo Antifungal Activity of ITZ–TF

#### 3.5.1. Evaluation of Symptoms Related to Dermal Fungal Infection

**Figure 3:**
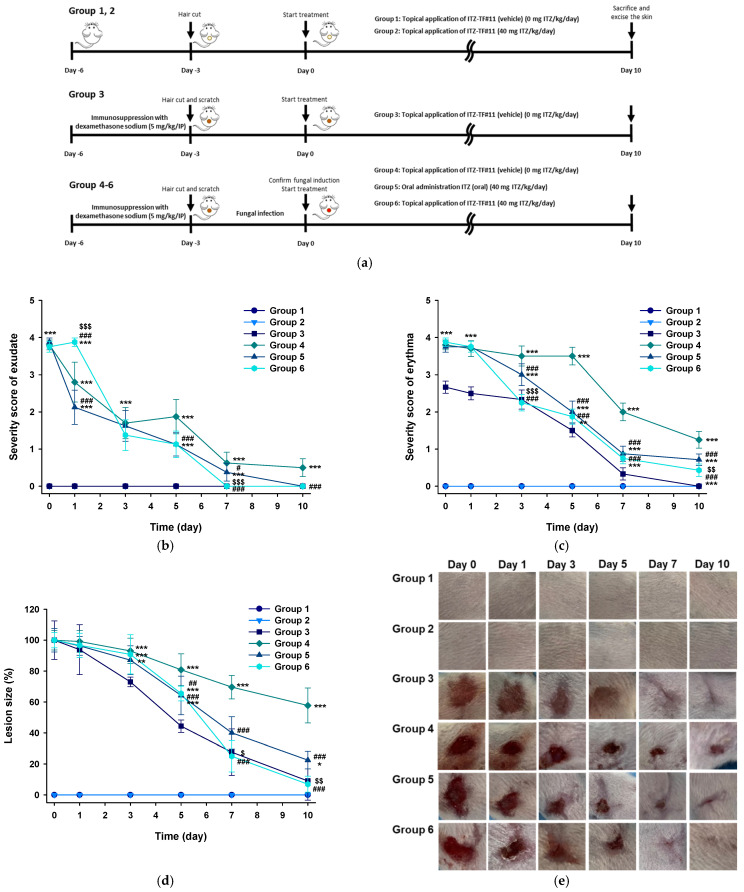
In vivo antifungal efficacies of once-daily oral or topical ITZ treatments in a mouse model of dermal *Candida albicans* infection. (**a**) Fungal infections and treatment schedules for once-daily oral and topical administrations of 40 mg/kg ITZ and ITZ–TF#11 (vehicle) or ITZ–TF#11 (equivalent to 40 mg/kg ITZ), respectively. (**b**) Exudates and (**c**) erythema from/on mouse skin lesions infected or not with *C. albicans* and treated with topical ITZ–TF#11 (vehicle) or ITZ–TF#11 or oral ITZ for 10 days. (**d**) Lesion sizes and (**e**) representative photographs of normal skin treated with ITZ–TF#11 (vehicle) (group 1) or ITZ–TF#11 (group 2); scratched skin treated with ITZ–TF#11 (group 3); and scratched and fungus-infected skin treated with ITZ–TF#11 (vehicle) (group 4), oral ITZ (group 5), and ITZ–TF#11 (group 6) for 10 days. Each value represents a mean ± standard deviation (*n* = 10 for each group). * *p* < 0.05, ** *p* < 0.01, *** *p* < 0.001 compared to scratched skin treated with ITZ–TF#11 (group 3). ^##^ *p* < 0.01, ^###^ *p* < 0.001 compared to scratched and fungus-infected skin treated with ITZ–TF#11 (vehicle) (group 4). ^$^ *p* < 0.05, ^$$^ *p* < 0.01, ^$$$^ *p* < 0.001 compared to scratched and fungus-infected skin treated with oral ITZ (group 5).

Responses during in vivo skin irritation and antifungal studies were evaluated based on lesion size, as well as the presence or absence of exudate, skin redness (erythema), and dry scaly skin (Figure 3a). Mice in groups 1 [normal skin treated with ITZ–TF#11 (vehicle)] and 2 [normal skin treated with ITZ–TF#11] did not show any obvious symptoms related to skin irritation, indicating suitable safety profiles of ITZ–TF#11 (vehicle) and ITZ–TF#11 in normal mouse skin. In addition, on day 0, the exudate severity scores for mice in groups 4 [fungal-infected skin treated with ITZ–TF#11 (vehicle)], 5 [fungal-infected skin treated with ITZ (oral)], and 6 [fungal-infected skin treated with ITZ–TF#11] were greater than the exudate severity score for mice in group 3 [scratched skin treated with ITZ–TF#11 (vehicle)]. This finding demonstrates successful fungal infection in the skin of mice in groups 4, 5, and 6 (Figure 3b). Similar severity scores of both exudate and erythema in groups 4, 5, and 6 on day 0 indicate comparable extents of fungal infection (Figure 3b,c). After 10 days of continuous treatment, mice in group 4 showed no obvious improvement in the infected area. In contrast, mice in groups 5 and 6 demonstrated 77.5% and 93.1% reductions in lesion size in the infected area (Figure 3d,e). Exudate symptoms were absent after 7 days of treatment among mice in group 6 (Figure 3b). Additionally, mice in group 6 showed a 165% lower mean erythema severity score, compared with mice in group 5 (Figure 3c). This enhanced treatment efficacy of ITZ–TF#11, with respect to symptoms related to superficial mycosis and lesion recovery at the infected site, may have been due to the immediate drug availability that permitted robust local action.

#### 3.5.2. Mycological Assay

**Figure 4:**
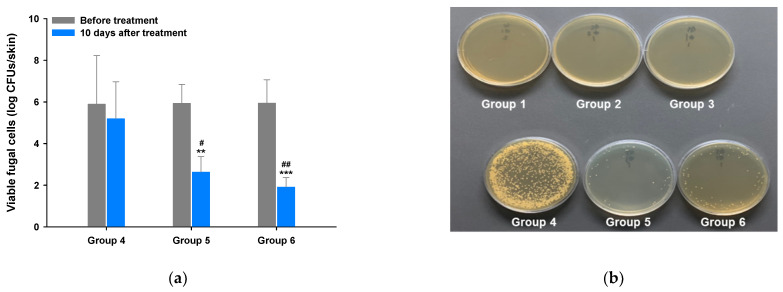
Mycological assay in full-thickness excisional normal, scratched, and scratched and fungal-infected mouse skins after once-daily treatment with ITZ–TF #11 (vehicle) (groups 1, 3, and 4) and ITZ–TF#11 (groups 2 and 6) based on 40 mg/kg ITZ or once-daily oral administration of 40 mg/kg ITZ (group 5) for 10 days. (**a**) Viable fungal cell counts in *C. albicans*-infected mouse skin lesions in groups 4, 5, and 6. Each value represents the mean ± standard deviation (*n* = 5). ** *p* < 0.01, *** *p* < 0.001 compared with initial log CFUs/skin section in each group. ^#^ *p* < 0.05, ^##^ *p* < 0.01 compared with log CFUs/skin section in group 4 after 10 days of treatment. (**b**) Representative photographs of viable fungal cells after 24 h incubation of excised normal, scratched, or scratched and fungal-infected mouse skins, following 10 days of treatment.

Next, we performed a mycological assay to investigate fungal viability in mouse skin during infection and after treatment with ITZ–TF#11 (vehicle) (group 4), ITZ (oral) (group 5), and ITZ–TF#11 (group 6). Groups 4, 5, and 6 exhibited similar mean viable fungal counts (log CFUs/skin section) on day 0, implying that all mice had been infected with a comparable number of fungi (Figure 4a). To directly assess antifungal efficacy, the CFUs of groups 4, 5, and 6 were measured after 10 days of continuous treatment. Figure 4b reveals the absence of fungal growth in groups 1 and 2, indicating the absence of microbial contamination during the mycological assay. The number of colonies did not significantly decrease in the mice of group 4. However, groups 5 and 6 exhibited 2.25- and 4.99-fold reductions in the log CFUs/skin section area, compared to the day 0 data, indicating complete removal of fungal cells from infected skin (Figure 4). Therefore, based on the antifungal response and the mycological assay findings, ITZ–TF#11 effectively treated superficial mycosis.

#### 3.5.3. Histological Evaluation of Skin

**Figure 5:**
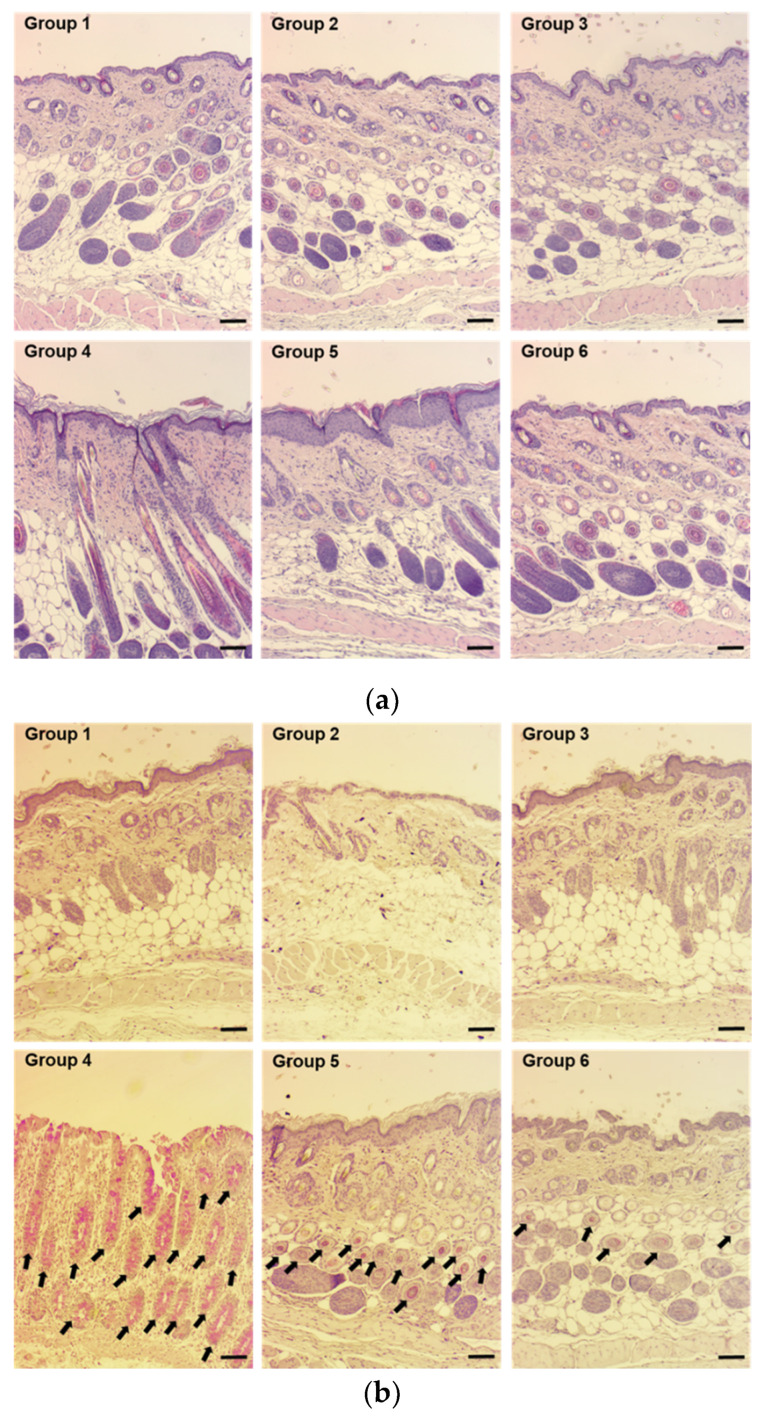
Representative light micrographs of tissue sections from normal, scratched, and scratched and fungal-infected mouse skin stained with (**a**) hematoxylin and eosin (H&E) and (**b**) periodic acid-Schiff (PAS) after 10 days of oral or topical ITZ treatment. PAS-positive reactions (red) were observed in the basement membrane of the epidermis, hair follicles, and sebaceous glands (black arrows). Scale bar = 100 μm.

To confirm the structural alteration and inflammation of skin from mice in groups 1 [normal skin treated with ITZ–TF#11 (vehicle)], 2 [normal skin treated with ITZ–TF#11], 3 [scratched skin treated with ITZ–TF#11(vehicle)], 4 [fungal-infected skin treated with ITZ–TF#11 (vehicle)], 5 [fungal-infected skin treated with ITZ (oral)], and 6 [fungal-infected skin treated with ITZ–TF#11] after 10 days of the indicated treatment, we performed histological examinations by staining with H&E. Groups 1, 2, and 3 did not exhibit any variations in skin structure, which supported the safety profiles of ITZ–TF#11 (vehicle) and ITZ–TF#11 (Figure 5a). However, group 4 demonstrated epidermal rupture and irregular granulation of fibroblast cells in the epidermis and dermis. In comparison with groups 4 and 5, skin from mice in group 6 demonstrated well-organized epidermis and dermis, together with thinner granulation tissue and denser mature collagen fiber bundles. Next, to confirm the cell viability among layers of skin, mouse skin sections from each group were examined using PAS staining. Skin from mice in groups 1, 2, and 3 did not demonstrate any obvious PAS markers related to fungi (Figure 5b). However, skin from mice in group 4 showed red-stained viable fungal cells (indicated by black arrows) in various layers of the skin. Conversely, skin from mice in group 6 demonstrated very few viable fungi cells, compared with skin from mice in groups 4 and 5. Overall, the enhanced efficacy of ITZ–TF#11 was presumably due to immediate drug availability at application sites that achieved maximal lysis of fungi, thereby promoting natural tissue proliferation and tissue remodeling, resulting in well-organized skin layers [34,35].

## 4. Conclusions

This study demonstrated that topical ITZ formulations prepared via the incorporation of ITZ into a biphasic system composed of volatile and non-volatile components substantially promoted drug solubility and stability during long-term storage. The optimal formulation (ITZ–TF#11) showed 132% greater skin permeability at 24 h, as well as 209% greater nail infiltration on day 7, compared with 1% ITZ in ethanol. In addition, permeability analysis showed that concentrations of drug deposition in skin and nail from ITZ–TF#11 were 539% and 230% greater than concentrations of drug deposition from 1% ITZ in ethanol, respectively. Moreover, ITZ–TF#11 achieved 323% greater fungal-infected skin lesion recovery, a 165% lower mean erythema severity score, and a 37% lower mean logarithm of viable fungal cells in skin at the treatment area, compared with skin treated with oral ITZ at the same dose. Thus, the long-term stability and improved permeability of ITZ–TF#11 resulted in the immediate availability of locally applied ITZ at the site of application, in comparison with ITZ (oral). The findings indicate that ITZ–TF#11 offers an effective alternative formulation against topical fungal infections.

## Figures and Tables

**Table 1:** Compositions of topical ITZ formulations.

Formulations (%, *w*/*w*)	ITZ	BHA	EDTA	Water	Benzyl Alcohol	HCl	1 M NaOH	Transcutol P	Cyclomethicone	Ethanol
ITZ in ethanol	1	0.1	0.0025	0.9975						97.900
ITZ–TF#1	1	0.1	0.0025	0.9975	10					87.900
ITZ–TF#2	1	0.1	0.0025	0.9975	20					77.900
ITZ–TF#3	1	0.1	0.0025	0.9975	30					67.900
ITZ–TF#4	1	0.1	0.0025	0.9975	30	0.121	1.12			66.659
ITZ–TF#5	1	0.1	0.0025	0.9975	30	0.242	2.13			65.528
ITZ–TF#6	1	0.1	0.0025	0.9975	30	0.484	4.12			63.296
ITZ–TF#7	1	0.1	0.0025	0.9975	30	0.484	3.64	10		53.776
ITZ–TF#8	1	0.1	0.0025	0.9975	30	0.484	3.48	15		48.936
ITZ–TF#9	1	0.1	0.0025	0.9975	30	0.484	3.46	20		46.044
ITZ–TF#10	1	0.1	0.0025	0.9975	30	0.484	4.64	15	5.00	47.160
ITZ–TF#11	1	0.1	0.0025	0.9975	30	0.484	4.68	15	8.22	39.156

**Table 2:** Group allocations.

Group	Immunosuppression	Scratching	Fungal Infection	Treatment	Administration Route	Dose of ITZ (mg/kg/day)
1				ITZ–TF#11 (vehicle)	Topical	
2				ITZ–TF#11	Topical	40
3	Immuno-suppression	Scratched		ITZ–TF#11 (vehicle)	Topical	
4	Immuno-suppression	Scratched	Infected	ITZ–TF#11 (vehicle)	Topical	
5	Immuno-suppression	Scratched	Infected	ITZ oral capsule	Oral	40
6	Immuno-suppression	Scratched	Infected	ITZ–TF#11	Topical	40

**Table 3:** Initial drug concentration, drug content after 1 month, and cumulative permeated drug from ITZ–TF through the artificial skin membrane.

Formulation	Initial Drug Concentration (mg/mL)	Drug Content after 1 Month (%) ^1^	Cumulative Permeated Drug (µg/mL) ^2^
ITZ in ethanol	0.32 ± 0.01	12.7 ± 0.69	0.244 ± 0.033
ITZ–TF#1	9.25 ± 0.01	38.9 ± 0.88	1.244 ± 0.120
ITZ–TF#2	9.33 ± 0.07	51.1 ± 0.30	2.560 ± 0.170
ITZ–TF#3	9.32 ± 0.95	58.4 ± 0.46	3.156 ± 0.012
ITZ–TF#4	9.24 ± 0.47	69.7 ± 0.70	3.250 ± 0.150
ITZ–TF#5	9.25 ± 0.20	80.0 ± 0.46	3.420 ± 0.105
ITZ–TF#6	9.38 ± 1.65	99.5 ± 0.54	3.814 ± 0.003
ITZ–TF#7	9.41 ± 0.21	99.8 ± 1.80	11.03 ± 1.465
ITZ–TF#8	9.36 ± 0.90	99.6 ± 0.15	15.59 ± 0.250
ITZ–TF#9	9.42 ± 0.11	99.8 ± 0.23	15.68 ± 0.941
ITZ–TF#10	9.40 ± 0.27	99.8 ± 1.21	24.75 ± 1.384
ITZ–TF#11	9.39 ± 0.21	99.9 ± 0.60	36.65 ± 1.045

^1^ Drug content after 1 month was calculated by defining the initial drug concentration as 100%. ^2^ Cumulative permeated drug concentrations from 1% ITZ dispersed in ethanol and ITZ–TFs were measured using an artificial skin membrane after 6 h of permeation. Each value represents the mean ± standard deviation (*n* = 6).

**Table 4:** Minimum inhibitory concentration (MIC) and minimum fungicidal concentration (MFC) of ITZ–TF#11 (vehicle), 1% ITZ in benzyl alcohol, and ITZ–TF#11.

Sample	MIC (µg/mL)	MFC (µg/mL)
ITZ–TF#11 (vehicle)	50.0	100
1% ITZ in benzyl alcohol	12.5	25
ITZ–TF#11	4.68	25

## Data Availability

Not applicable.
